# GAEDGRN: reconstruction of gene regulatory networks based on gravity-inspired graph autoencoders

**DOI:** 10.1093/bib/bbaf232

**Published:** 2025-05-26

**Authors:** Pi-Jing Wei, Huai-Wan Jin, Zhen Gao, Yansen Su, Chun-Hou Zheng

**Affiliations:** Information Materials and Intelligent Sensing Laboratory of Anhui Province, Institute of Physical Science and Information Technology, Anhui University, 111 Jiulong Road, Hefei 230601, Anhui, China; School of Artificial Intelligence, Anhui University, 111 Jiulong Road, Hefei 230601, Anhui, China; The Key Laboratory of Intelligent Computing and Signal Processing of Ministry of Education, School of Computer Science and Technology, Anhui University, 111 Jiulong Road, Hefei 230601, Anhui, China; School of Artificial Intelligence, Anhui University, 111 Jiulong Road, Hefei 230601, Anhui, China; School of Artificial Intelligence, Anhui University, 111 Jiulong Road, Hefei 230601, Anhui, China

**Keywords:** gene regulatory networks, graph convolutional network, gravity-inspired graph autoencoder, gene importance, random walk regularization

## Abstract

Reconstructing high-resolution gene regulatory networks (GRNs) based on single-cell RNA sequencing data provides an opportunity to gain insight into disease pathogenesis. At present, there are a large number of GRN reconstruction methods based on graph neural networks, and they can obtain excellent performance in GRN inference by extracting network structure features. However, most of these methods fail to fully exploit the directional characteristics or even ignore them when extracting network structural features. To this end, a novel framework called GAEDGRN is proposed based on gravity-inspired graph autoencoder (GIGAE) to infer potential causal relationships between genes. Among them, GIGAE can help us capture the complex directed network topology in GRN. Additionally, due to the uneven distribution of the latent vectors generated by the graph autoencoder, a random walk-based method is used to regularize the latent vectors learnt by the encoder. Furthermore, considering that some genes in GRN usually have a significant impact on biological functions, GAEDGRN designs a gene importance score calculation method and pays attention to genes with high importance in the process of GRN reconstruction. Experimental results on seven cell types of three GRN types show that GAEDGRN achieves high accuracy and strong robustness. Moreover, a case study on human embryonic stem cells demonstrates that GAEDGRN can help identify important genes.

## Introduction

Gene regulatory networks (GRNs) represent a causal regulatory relationship between transcription factors (TFs) and their gene targets [1]. The regulation of target genes by TF not only responds to environmental signals, but also determines the identity and function of cells [2]. GRN plays an important role in cell differentiation, development, and disease progression [3], therefore, reconstructing the GRN is essential for further study of tissues and functions in the context of health and disease [4].

The traditional experiment-based approaches focus more on functional pathways than on reconstructing the entire network [5], which is time-consuming and labor-intensive. Thus, a great number of computational methods have been developed. Because of the development of biotechnology (such as high-throughput measurement of gene expression abundance), a large amount of gene expression data has been accumulated.

Single-cell RNA sequencing (scRNA-seq) technology, as opposed to bulk RNA sequencing (bulk RNA-seq), reveals biological signals in the gene expression profiles of individual cells without the need to purify each cell type [6]. Therefore, there is an urgent need to develop accurate tools to infer cell type-specific GRN based on scRNA-seq data, which is of great significance for understanding the pathogenesis of disease.

Currently, methods for inferring GRN from scRNA-seq data can be divided into unsupervised and supervised methods. Unsupervised approaches discover latent patterns from scRNA-seq data and infer regulatory relationships between genes based on statistical or computer techniques [7–12]. Supervised learning methods [13–19] construct training sets with known GRN as labels and scRNA-seq data as features, and then use deep learning techniques to learn regulatory knowledge from the training set to predict potential GRN. Compared with unsupervised learning methods, supervised models [13] tend to have higher accuracy because they can learn prior knowledge from labels and identify subtle differences between training samples through deep learning technologies. We can broadly categorize supervised methods into two groups based on the types of features they extract: one group concentrates on extracting gene expression features, while the other focuses on learning information related to network structures.

The first type of methods mainly uses convolutional neural network (CNN), recurrent neural network (RNN), Transformer, and other technologies to extract the gene expression characteristics of TF–gene pairs, and then determines the regulatory relationship between TF and target genes through classification, such as CNNC [13], TDL [20], DGRNS [18], and STGRNS [21]. CNNC [13] and TDL [20] convert the gene expression data of gene pairs into images and then use CNN, RNN, and other technologies to extract features. The former reconstructs GRN based on static scRNA-seq data, and the latter reconstructs GRN based on time-series scRNA-seq data. However, the generation of image data in these methods not only generates unexpected noise, but also hides some of the original data features. In addition, the training process of these methods is time-consuming, especially for large datasets. Considering that the gene expression data are a one-dimensional sequence, DGRNS [18] and STGRNS [21] use one-dimensional CNNs, RNNs, and Transformer to directly extract gene expression features from the gene expression data, and show excellent prediction performance on the benchmark dataset, which solved the shortcomings of CNNC and TDL. However, these methods ignore the complex network structure characteristics of GRN.

The second type of methods focuses on the complex network topology characteristics of GRN. For example, GNE [22] is a gene network embedding method that uses network topology and gene expression profile data to predict gene interactions through multilayer perceptron (MLP). However, due to the limited ability of MLP to learn the structural features of the network, GNE has encountered difficulties in fully understanding gene topology. In recent years, graph neural network (GNN)-based methods have been widely used in link prediction tasks, which learn the embedding of nodes by aggregating information of neighbors. As the task of reconstructing GRN can be regarded as link prediction problem, some researchers have used GNNs to infer GRN from scRNA-seq data, such as GENELink [16] and DeepTFni [23]. GENELink [16] uses the gene expression data as node feature, then leverages the graph attention network (GAT) to perform message passing on the incomplete prior network, and finally captures the network structure features of GRN, and predicts the regulatory relationship between TF and target genes based on this. However, GENELink does not consider the directionality of GRN when considering the structural features of the graph. DeepTFni [23] first uses single-cell ATAC-seq data to obtain a priori GRN, and then employs a variational graph autoencoder (VGAE) to reconstruct the GRN. However, the VGAE model used by DeepTFni can only predict undirected GRN. In addition, the method ignores the fact that some genes are both regulatory and target genes in biological reality. In summary, these methods ignore the directionality of the edges in the GRN when extracting network structure features, thereby impeding the prediction performance of GRN inference.

In order to solve the above problems, we propose a supervised model GAEDGRN for inferring GRN from scRNA-seq, which can effectively consider the directed network topology characteristics and gene expression characteristics in GRN. Considering that GRN is a complex directed graph, gravity-inspired graph autoencoder (GIGAE) [24] is introduced to effectively extract the directed network structure features of GRN and infer directed GRN. In addition, considering that some important genes in GRN will have a significant impact on biological processes or functions, GAEDGRN designs a method PageRank^*^ to calculate the importance score of genes and applies it to the encoding and decoding process of GIGAE. Finally, the distribution of embedding vectors generated by GAE is often uneven, which may result in poor embedding effect, so GAEDGRN adopts random walk regularization to standardize the latent embedding vectors of genes learned by GIGAE. Experimental results on seven cell types and three GRN types show that the GAEDGRN model could effectively improve the recognition performance and reduce the training time.

Overall, our main contributions are summarized as follows:

- GAEDGRN adopts GIGAE to learn the structure information of the complex directed network and gene expression characteristics in the GRN, which improves the prediction of causal regulatory relationship between genes.
- The improved PageRank^*^ algorithm is proposed to calculate the importance score of genes, which makes the model pay more attention to important genes when learning gene characteristics and making causal inferences by focusing on the out-degree of genes.
- The random walk regularization module is introduced to standardize the learning of gene latent vectors by GIGAE, so that the latent vectors are evenly distributed and the effect of embedding is improved.
- Experimental results show that GAEDGRN successfully optimizes the training process of gene features, significantly improves the performance of the model, and effectively reduces the training time, which makes it a valuable tool in the GRN prediction task.

## Materials and methods

### Overview of GAEDGRN

**Figure 1:**
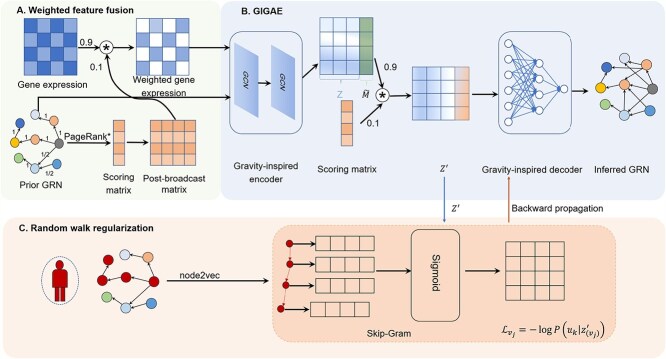
Overview of the GAEDGRN framework. (A) Weighted feature fusion calculates the gene importance score based on PageRank^*^, and then performs feature fusion with the gene expression matrix to obtain a weighted feature fusion matrix. (B) GIGAE: the prior GRN and weighted feature fusion matrix are input to GIGAE to obtain a vector of (*d*+1) dimensions for each gene through an encoder in which the first $d$ dimensions represent the potential representation of the gene, and the last dimension is represented by the mass parameters $\tilde{M}$. $\tilde{M}$ is characteristically fused with the gene importance score to obtain the last dimension of the latent vector $Z^{\prime}$, and finally the unknown gene regulatory relationship is inferred by the decoder. (C) Random walk regularization: random walk learns a series of node sequences, and sends these nodes into the Skip-Gram module to obtain a subset of structurally similar nodes (the number is consistent with the Skip-Gram window size), and the node features are extracted from $Z^{\prime}$. During training, the goal is to maximize the probability of a node appearing with nodes that are present in the window. The objective is to minimize the loss function, which involves passing gradient information from a Skip-Gram module to another module B.

GAEDGRN is a supervised deep learning framework for inferring GRN based on scRNA-seq gene expression data and a prior GRN. GAEDGRN focuses on training the latent embedding representation of genes and reconstructing directed GRN in the case of concern for important genes. The proposed framework (Fig. 1) consists of three parts: (A) Weighted feature fusion. In this module, an improved method PageRank^*^ based on the PageRank method is first proposed, and then the gene importance score is calculated based on it. Next, the importance score is fused with the features of the gene expression matrix that make the encoder pay more attention to important genes. (B) GIGAE. GIGAE is used to extract the structural features of directed networks, and the importance scores of genes are considered while reconstructing directed GRN. (C) Random walk regularization. The random walk can capture the local topology of the network. Then the node access sequence obtained by the random walk and the potential embedding $Z^{\prime}$ of the gene after feature fusion in GIGAE are used to minimize the loss function in the Skip-Gram module. In this process, the gradient propagation mechanism plays a key role in ensuring that the gradients computed from the loss function can be fed back into the potential embeddings learned by GIGAE, so that these embeddings can be normalized.

### Weighted feature fusion

#### Gene importance scores calculation based on PageRank^*^

PageRank algorithm [25] is designed to assess the importance of web pages, and it is widely used in search engine optimization to evaluate the effectiveness of web page optimization. It calculates the importance of a page based on the in-degree of the page and the importance of the neighbor pages linked to this page, and the quantitative and qualitative assumptions of the PageRank algorithm are shown in Supplementary Fig. S1.

**Figure 2:**
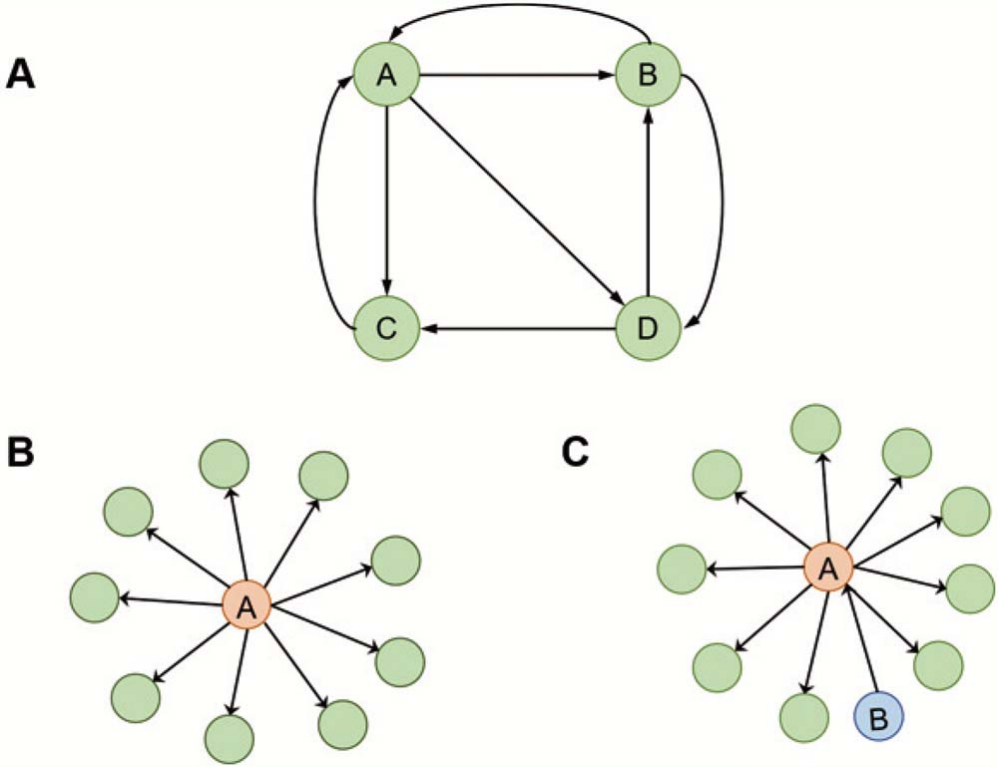
Schematic diagram of PageRank-related concepts. (A) Node link graph. (B) quantitative hypothesis: in a GRN, genes of high importance are characterized by their ability to regulate many other genes, as does gene $A$. (C) Quality assumption: if gene $A$ is an important gene, and $B$ regulates $A$, it means that $B$ is also an important gene.

In this study, we hypothesize that genes that regulate more other genes are of high importance. Therefore, the focus is on the out-degree of nodes rather than their in-degree. We propose qualitative and quantitative assumptions based on the assumptions of PageRank (see the Supplementary Fig. S1) and adopt an improved method PageRank^*^. The quantity hypothesis of PageRank^*^ is defined as follows: in GRN, such as shown in Fig. 2(B) for gene $A$, a gene that regulates many genes is considered to be an important gene. In the literature [26, 27], nodes with a degree greater than 7 are considered hub genes. However, in this paper, nodes with a degree of 7 or greater are considered important genes. The quality hypothesis is defined as follows: if a gene (gene $B$ in Fig. 2(C)) regulates an important gene (gene $A$), then the importance of that gene (gene $B$) is also high.

According to the assumption of PageRank^*^, $B$ and $C$ in Fig. 2(A) both point to $A$, so the probability that gene $A$ may be regulated by $B$ and $C$ is 1/2. Therefore, the transition matrix M^*^ for PageRank^*^ is defined as follows: 


(1) \begin{align*}& M^{*}=\left[\begin{array}{@{}cccc@{}} 0 & 1 / 2 & 1 / 2 & 1 / 2 \\ 1 / 2 & 0 & 0 & 1 / 2 \\ 1 / 2 & 0 & 0 & 0 \\ 0 & 1 / 2 & 1 / 2 & 0 \end{array}\right]\end{align*}

Initially, by default, the importance score *v* of each gene is the same, and we bring the obtained transfer matrix M^*^ into the PageRank^*^ formula to calculate the importance score *$ v^\prime $* of each gene: 


(2) \begin{align*}& v^{\prime} = \beta M^{*} v + \frac{(1 - \beta)e}{n},\end{align*}

where $v$ is score vector of current iteration, while $v^{\prime}$ is the score vector of PageRank^*^ after current iteration. $\beta $ is the damping factor (a real number between 0 and 1, default value $\beta =0.85$ based on experience), representing the probability that a gene regulates another gene through predefined regulatory links, while $1 - \beta $ denotes the probability of random regulation. $M^{*}$ is the transition probability matrix encoding pairwise regulatory relationships between genes, where the element $M^{*}_{ij}$ specifies the probability of gene $i$ being regulated by gene $j$. $e$ is a vector of $n*1$ with all elements being 1, representing random regulation scenarios. $n$ is the total number of genes.

#### Feature fusion strategy

In order to make the encoder pay more attention to the features of important genes in feature extraction, we incorporate the feature weight of genes. The importance score of each gene is calculated from the prior GRN by the PageRank^*^ algorithm and denoted as $PageRank^{*}(G)$. To enable effective feature fusion, the importance score of each gene is broadcast to match the dimension of the gene expression matrix *T*, formally denoted as $PBM(\mathit{G})$. Next, we integrate *T* with $PBM(\mathit{G})$ in a certain proportion to obtain a new weighted feature fusion matrix *X*. 


(3) \begin{align*}& X = \alpha * \mathit{PBM}(G) + (1 - \alpha) * T,\end{align*}

where hyperparameter *$\alpha $* is set to 0.1 (see the Parameter analysis section).

In the GIGAE framework, the higher the value of the mass parameters $\tilde{M}$ (see the Gravity-inspired graph autoencoder section), the higher the probability of connections between nodes. Here, $\tilde{M}$ denotes the last dimension of the genetic latent embedding vectors learned by the GIGAE framework. In order to reflect the relationship that genes with higher PageRank^*^ values are more likely to be connected to other genes, we conducted feature fusion between $PageRank^{*}(\mathit{G})$ and the corresponding mass parameters $\tilde{M}$. This fusion process ensures that genes of higher importance play a greater role in judging linkages with other genes. 


(4) \begin{align*}& \tilde{M}^{\prime} = \alpha* \mathit{PageRank^*}(G) + (1 - \alpha) * \tilde{M}\end{align*}

### Gravity-inspired graph autoencoder

GAE, as an advanced method for link prediction, has emerged in recent experiments [28, 29]. It can automatically learn low-dimensional feature representations of graph data, which can help to capture the complex relationships between nodes. However, using traditional GAE leads to an undirected network. In this study, we adopt GIGAE [24], which is inspired by the Newton’s theory of universal gravitation to improve the traditional GAE, to extract the structural features of directed networks and predict directed regulatory links between genes.

GIGAE consists of two parts: encoder and decoder. The encoder maps the GRN into a low-dimensional embedding space to generate latent gene embedding vectors and mass parameters, where a higher mass parameter value indicates that the corresponding gene is more likely to be regulated by other genes. The decoder computes regulatory association scores for gene pairs using the latent embedding vectors and mass parameters produced by the encoder, thereby achieving GRN prediction. In order to explain the procedure clearly, we firstly introduce the theory of universal gravitation, and then introduce the encoder and decoder.

According to the law of universal gravitation, if there are two objects with masses $m_{1}$ and $m_{2}$, the gravitational force $F$ between them is defined as in Equation 5. 


(5) \begin{align*}& F = \frac{G m_{1} m_{2}}{r^{2}},\end{align*}

where *G* is the gravitational constant, and $r$ is the distance between the two objects. And the acceleration $a_{1 \rightarrow 2}$, which means the acceleration of object 1 toward object 2, due to gravity is 


(6) \begin{align*}& a_{1 \rightarrow 2} = \frac{F}{m_{1}} = \frac{G m_{2}}{r^{2}}.\end{align*}

Similarly, the acceleration $a_{2 \rightarrow 1}$ of object 2 toward 1 is 


(7) \begin{align*}& a_{2 \rightarrow 1} = \frac{F}{m_{2}} = \frac{G m_{1}}{r^{2}}.\end{align*}

If $m_{1}> m_{2}$, then $a_{1 \rightarrow 2} < a_{2 \rightarrow 1}$. Salha *et al*. [24] have applied it to node representation and proposed an assumption that the mass parameter $m_{j}$ could capture the tendency of node $j$ to attract other nodes in directed graph, which means these nodes point to $j$. Therefore, the direction from node $i$ to node $j$ can be represented by acceleration 


(8) \begin{align*}& a_{i \rightarrow j} = \frac{G m_{j}}{r^{2}}.\end{align*}

As GRN is a directed graph, this assumption and application provide an effective tool for analyzing the regulatory relationship of gene pairs in GRN. Here, we assume that $z_{i}$ represents a latent vector with dimensions $d$ for gene $i$, and a mass parameter $m_{i}$ is a mass parameter for each gene $i$. Applying Newton’s equation to the latent space, the acceleration in Equation 8 can be used as an indicator of the potential that node $i$ point to node $j$. In Equation 8, the larger the mass of node $j$, the more likely it is that node $i$ points to node $j$. And the denominator distance $r$ is abstracted as $r^{2} = \left \| z_{i} - z_{j} \right \|_{2}^{2}$, which means that the closer the latent vectors are, the more likely the nodes are to connect. According to GIGAE [24], for convenience, the logarithmic transform of $a_{i \rightarrow j}$ (denoting as $log a_{i \rightarrow j}$) is used instead of directly using $a_{i \rightarrow j}$.

In the encoder part, it contains two layers of GCN and the input data are adjacency matrix and gene expression matrix. After two layers of GCN, the output of each node is a vector with ($d$+1) dimensions, in which the first $d$ dimensions correspond to the latent vector $z_{i}$ of the gene $i$, and the last dimension represents the mass parameter. The output is as follows: 


(9) \begin{align*}& (Z, \tilde{M}) = GCN(\tilde{A}, X),\end{align*}

where *Z* is the latent representation matrix of dimensions n^*^d, with each row $z_{i}$ representing the latent embedding of a gene. $\tilde{M}$ is the mass parameters vector with $n$^*^1 dimension. Then the $\tilde{M}$ is fused with $PageRnak^{*}(G)$ to obtain $\tilde{M}^{\prime}$ according to feature fusion strategy. And $\tilde{A} = D_{\textrm{out}}^{-1} (A + I)$ is the out-degree normalized version of the adjacency matrix *A* for GRN.

In the decoder part, $\tilde{M}^{\prime}$ and $Z$, together with the predefined logarithmic acceleration and a sigmoid activation function, are employed to reconstruct adjacency matrix $\hat{A}$ as Equation 10. 


(10) \begin{align*} \hat{A}_{ij} &= \sigma \big( \log a_{i \rightarrow j} \big)\nonumber \\ &= \sigma \left( \log \frac{G m_{j}}{r^{2}} \right) \nonumber \\ &= \sigma \big( \log G m_{j} - \log r^{2} \big)\nonumber \\ &= \sigma \Big( \tilde{m}^{\prime}_{j} - \log \left\| z_{i} - z_{j} \right\|_{2}^{2} \Big), \end{align*}

where $\tilde{m}^{\prime}_{j}$ represents the result of feature fusion between the last dimension of the latent vector of gene $j$ and its gene importance score, which is the model’s estimate of $\tilde{m}^{\prime}_{j} = logGm_{j}$. Since $\tilde{m}^{\prime}_{i}$ and $\tilde{m}^{\prime}_{j}$ are distinct values, the gravitational formula is employed to determine the directionality between genes $i$ and $j$ in the GRN.

### Random walk regularization

Random walk regularization makes the distribution of potential vectors of structurally similar genes more uniform and more similar [30]. Applying this to the decoder of GIGAE makes $\left \| z_{i} - z_{j} \right \|_{2}^{2}$ smaller, making it more likely for two structurally similar genes to be connected.

First, we use the node2vec algorithm [31] for random walk, which combines the advantages of depth-first search and breadth-first search to flexibly explore the network structure. The pseudocode of the node2vec algorithm is shown in the Supplementary Table S1.

**Figure 3:**
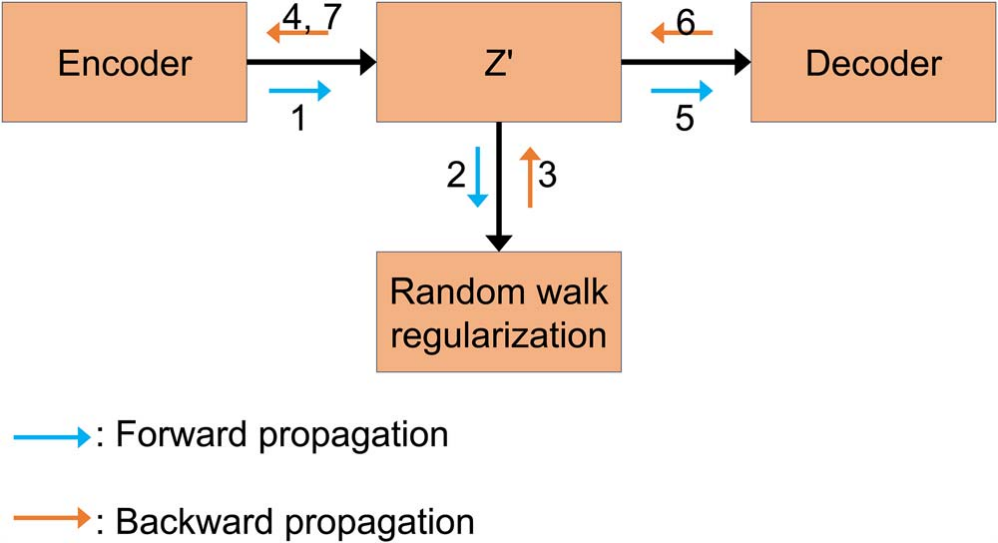
Random walk regularization and GIGAE gradient propagation pathways. The encoder of GIGAE learns a latent gene embedding $Z^{\prime}$ (step 1), which is forward propagated to the random walk regularization module (step 2). During this process, as the loss function is minimized, gradient backpropagation constrains $Z^{\prime}$ (step 3). The updated $Z^{\prime}$ is then backward propagated to both the encoder (step 4) and decoder of GIGAE (step 5), thereby further enhancing the similarity between the latent embeddings of two structurally similar genes (steps 6 and 7).

As shown in Fig. 1(C), we generate a series of node access sequences using the node2vec algorithm. These sequences are then used as input to a Skip-Gram model, which is used to predict the context nodes of each node. Specifically, we multiply the eigenvectors of a node by the transpose of the eigenvectors of its context node to get a matrix. This matrix is then processed by the sigmoid function so that we can compute the loss function and minimize it. In the process of minimizing the loss function, the calculated gradient is passed back to the GIGAE model, constraining the gene embeddings generated by it and thus regularizing encoder learning of gene latent vectors. The pseudocode for random walk regularization in the Supplementary Table S2. In the process of the descent of the random walk loss function, the propagation path of the gradient is shown in Fig. 3.

## Results

### Datasets

We demonstrate the prediction performance of GAEDGRN on benchmark datasets from BEELINE [32]. These datasets comprise scRNA-seq data of seven cell types and three kinds of networks. The seven cell types are human embryonic stem cells (hESCs), human mature hepatocytes (hHEP), mouse dendritic cells (mDCs), mouse embryonic stem cells (mESCs), mouse erythroid hematopoietic stem cells (mHSC-E), mouse granulocyte-monocyte lineage hematopoietic stem cells (mHSC-GM), and mouse hematopoietic stem cells with lymphoid lineage (mHSC-L). The scRNA-seq data of the above cell types can be downloaded from Gene Expression Omnibus using the following accession numbers: GSE81252 (hHEP), GSE75748 (hESC), GSE98664 (mESC), GSE48968 (mDC), and GSE81682 (mHSC), respectively. The three kinds of ground-truth networks are cell-type-specific ChIP-seq networks [33–35], nonspecific ChIP-seq networks [36–38], and functional interaction networks from the STRING database [39]. Each cell type can correspond to three types of networks.

Taking into account the large amount of raw scRNA-seq data and the high redundancy, GENELink [16] filtered out low-expressed genes during the processing steps and focused on genes with significant variability in expression. Therefore, in this study, we used the datasets that have been processed by GENELink (Supplementary Table S3). The dimension of the feature matrix *X* is *N*$\times $*M*, where $N$ represents the number of genes and $M$ represents the number of cells. We selected TFs and the top 500 and 1000 genes with the greatest expression variability (denoted as TFs+500 and TFs+1000) to construct 42 different datasets.

### Experimental setting and training strategy

In this work, the known TF–gene pairs in the ground-truth networks from cell-type-specific ChIP-seq networks, nonspecific ChIP-seq networks, and STRING were called positive samples and labeled as 1. TF–gene pairs absent from the ground-truth network were designated as negative samples and labeled 0. We randomly selected 85% of the known gene pairs as the positive edges of the training set and constructed a training adjacency matrix. The remaining 15% were used as the positive edges of the test set (10%) and the validation set (5%), respectively, and the corresponding number of negative samples were selected as the negative edges of the test set and the validation set. Our model was trained on the training set and used the validation set for parameter optimization and iterative tuning. This iterative optimization process allowed us to fine-tune the performance of the model and enhanced its ability to capture potential regulatory dependencies in the gene network. We used average area under the receiver operating characteristic curve (AUROC) and area under the precision-recall curve (AUPRC) to measure the performance of GAEDGRN; detailed explanations of AUROC and AUPRC can be found in the Supplementary text. For the training of the GAEDGRN model, we set a training cycle of 100 iterations and used a learning rate of 0.001.

### Parameter analysis

**Figure 4:**
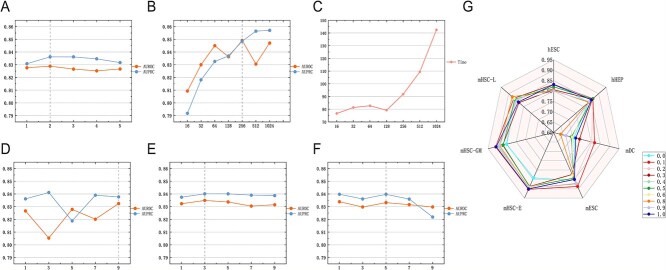
Parameter sensitivity analysis. (A) The influence of different numbers of GCN layers on the results. (B) The performance of different dimensions of GCN on the results. (C) The time required for GCN of different dimensions; the unit for the vertical axis is seconds. (D) The influence of different length of random walk on the results. (E) The influence of different window size of Skip-Gram on the results. (F) The effect of different number of random walk on each node on the results. (G) The effect of different feature fusion parameters on the results. In the study, Figures A, B, D, E, and F utilize both area under the receiver AUROC and AUPRC to measure the average performance among seven datasets, while Figure G solely employs the AUROC metric.

The performance of our model is affected by several key parameters, including the number of layers ($L$) and dimension ($D$) of the GCN in the encoder, the length of the walk length, the window size and the number of walks, and the ratio of the importance score of the gene to the gene expression matrix/mass parameters fusion (*$\alpha $*) at the time of feature fusion. To assess the model’s effectiveness in predicting GRN, we conducted an evaluation on cell-type-specific GRN reconstruction for seven cell types and the results were shown in Fig. 4.

In the GIGAE encoder, the number of layers ($L$) plays a crucial role in determining the characteristics of adjacent genes. To assess the effect of this parameter, we systematically adjusted $L$ from 1 to 5 in steps of 1. The analysis in Fig. 4(A) shows that setting $L$ to 2 yields the highest average AUROC and mean of AUPRC values. Interestingly, when more layers are used, a decreasing value of AUROC is observed, possibly due to overfitting and smoothing issues. Next, we investigated the effect of the representation dimension ($D$) in the model by assigning $D$ the following values: 16, 32, 64, 128, 256, 512, and 1024. The results in Fig. 4(B) show that the model performs the best when the value of $D$ was 256 or 1024. The overall trend in Fig. 4(C) shows that the higher the $D$ value, the longer the runtime. In order to balance the performance and time complexity, we chose a value of 256 for dimension ($D$). In the random walk part of our study, we focused on the impact of three key parameters on the model’s performance: node length, window size, and the number of the random walk per node. Specifically, we investigated the effects of varying these parameters within the range of 1 to 9, with increments of 2 (i.e. 1, 3, 5, 7, 9), to determine how different parameter values influence the model’s ability to capture regulatory dependencies in the gene networks. Based on the results in Fig. 4(D–F), we chose the node length, window size, and number of walks for random walk to be 9, 3, and 5, respectively. In the feature fusion section, we investigated the effect of adding gene importance scores and selected the feature fusion ratio *$\alpha $* from 0 to 1 in steps of 0.1. As shown in Fig. 4(G), the model performs the best with an *$\alpha $* of 0.1.

The GNN architecture consists of two hidden layers, each with a size of 256. The ratio of feature fusions was set to 0.1, where the weight of the gene importance score was 0.1 and the weight of the feature/mass parameters was 0.9. The length of the node for the random walk was 9, the size of the Skip-Gram window was 3, and the number of random walk on each node was 5. In the subsequent part, we conducted an analysis of the parameters of the GCN-based graph encoder, including the number of layers ($L$), the dimension ($D$), the optimization of random walk parameters, and the feature fusion ratio (*$\alpha $*). All results were reported based on the average values obtained from 10 independent tests. To ensure fairness and consistency in the evaluation, we used the same set of training and validation for all supervised methods. This approach allowed us to compare the performance of each model on the same test set, eliminating the bias that can come with different data segments.

### Performance on benchmark datasets

#### Benchmark methods

- CNNC [13]: after converting the gene expression data of the TF–target gene pair into images, a deep CNN is used to extract gene expression features and infer GRN.
- DGRNS [18]: this method combines an RNN for temporal feature extraction and a CNN for spatial feature extraction to infer GRN.
- STGRNS [21]: this method introduces a gene expression motif technology, which encodes gene pairs into contiguous daughter vectors, and then they are input into Transformer for gene expression feature extraction and GRN inference.
- GENELink [16]: it proposes a GAT method to infer potential GRN.
- GNE [22]: it proposes an MLP method to encode gene expression profiles and network topologies to predict gene dependence.
- DeepTFni [23]: this method uses VGAE to infer GRN from scATAC-seq data.

For consistency and comparability, we use the default parameters provided in the original implementation of all baseline methods.

#### Performance comparison of GAEDGRN and benchmark methods

**Figure 5:**
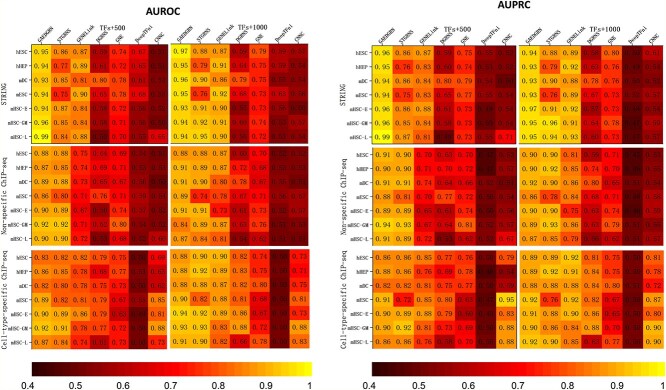
Experimental results of GAEDGRN compared with existing methods. Figures (A) and (B) illustrate the performance of GRN prediction in the AUROC and AUPRC metrics, respectively. The AUROC and AUPRC values in the heatmap are the average of 10 independent test calculations for each data set.

In order to better evaluate the accuracy of GAEDGRN for GRN inference, we conducted 10 independent tests on 42 datasets (Supplementary Table S3) and calculated average AUROC and AUPRC. The datasets were divided into a training set (85%), a validation set (5%), a test set (10%), and conducted experiments according to the training and testing strategies described in the first section of this chapter. The performance of GAEDGRN and benchmark methods is shown in Fig. 5, and it shows that GAEDGRN outperforms the vast majority of benchmark methods in terms of AUROC and AUPRC. Specifically, GAEDGRN achieves the best prediction performance on 90.5% (38 out of 42) and 88.1% (37 out of 42) benchmark datasets in terms of AUROC and AUPRC metrics, respectively. Compared with the second-best method (STGRNS), we have drawn the following conclusions. Firstly, it improves the AUROC at least 4% for about 38.1% (16 out of 42) of the benchmark datasets. Secondly, the AUROC metric of GAEDGRN outperforms STGRNS on 71.4% (30 out of 42) of the benchmark datasets, and the AUPRC of GAEDGRN exceeds STGRNS on 66.7% (28 out of 42) of the benchmark datasets. Although the STGRNS model uses more popular Transformer method, it only uses the expression characteristics of genes. In contrast, GAEDGRN considers the network structure of GRN while considering the gene expression characteristics, which also makes it superior to STGRNS on the vast majority of datasets. Similarly, the prediction performance of GAEDGRN is higher than that of CNNC and DGRNS. Compared with GENELink and DeepTFni, the inference accuracy of our model in the vast majority of datasets on GRN is higher than them because our model takes into account the direction of the GRN, which is very important for GRN reconstruction.

### Ablation experiments

**Figure 6:**
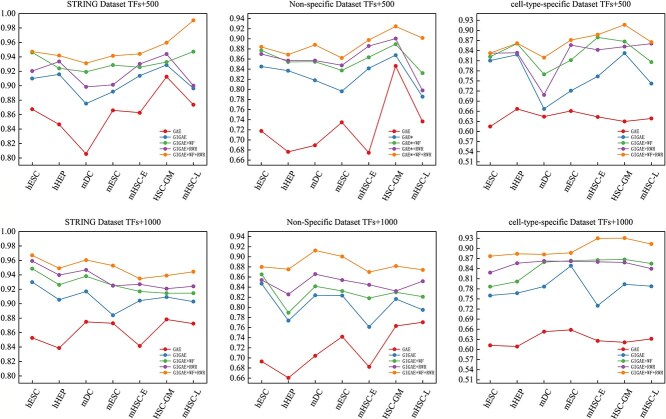
Ablation results. GAE stands for using traditional GAE, GIGAE, WF stands for weighted feature fusion, and RWR stands for random walk regularization.

Ablation experiments are performed to analyze the effectiveness of each module. The experiments include traditional GAE, GIGAE, GIGAE with weighted feature fusion (GIGAE+WF), GIGAE with random walk regularization (GIGAE+RWR), and GAEDGRN (GIGAE+WF+RWR). The experimental results on different datasets are shown in Fig. 6. It can be found that each module in GAEDGRN has an important impact on GRN inference.

As can be seen from Fig. 6, the results of GIGAE are higher than those of traditional GAE regardless of the cell type, indicating that considering the GRN direction can effectively improve the prediction performance of the model. One of the most critical aspects is that GIGAE can help us obtain directed GRNs, which do not ignore the fact that some target genes are also regulators compared with previous transcriptional regulatory networks.

Figure 6 clearly demonstrates that the performance metrics of the GIGAE+WF+RWR model consistently outperform those of the GIGAE+RWR model across all datasets, suggesting that incorporating gene importance significantly enhances the predictive accuracy of the model. There are some reasons for this improvement. First of all, it can incorporate the importance score of genes into the input trait, which makes us pay more attention to the characteristics of the gene with high importance. Then, after the gene data pass through the GIGAE decoder, gene embedding matrix is obtained. According to the principles of the GIGAE decoder, the last dimension of this embedding matrix represents the mass parameters. The greater the value of this parameter, the higher the likelihood that the two genes are connected. The mass parameters of the last dimension are fused with the gene importance score, thereby ensuring that the higher the importance score of the gene, the more likely it is to be linked by other genes.

Similarly, it is clear in Fig. 6 that the GIGAE+WF+RWR model outperforms the GIGAE+WF model across all datasets, indicating the effectiveness of the random walk regularization module in improving model prediction accuracy. In order to enable the GIGAE encoder to obtain a more uniform distribution of gene potential vectors and achieve excellent embedding effects, we introduced the random walk regularization module. As shown in Supplementary Fig. S2(A), the latent vector distribution of genes is more dense and uneven without the addition of the random walk regularization module. As shown in Fig. S2(B) of the Supplementary Figures, the potential vector distribution of genes is more uniform with the addition of the random walk regularization module. The results of the ablation experiment show that the addition of random walk regularization also improves the effect of embedding.

### Stability analysis

**Figure 7:**
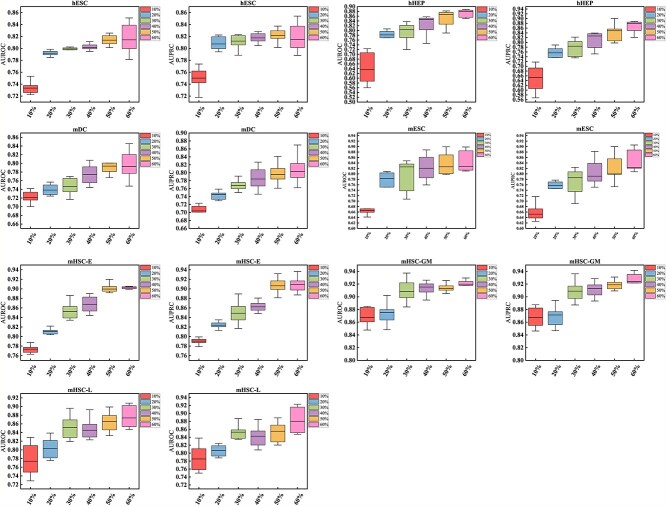
The performance of GAEDGRN with a wide range of training set size from 10% to 60% on the all TFs+500 datasets with cell-type-specific ChIP-seq networks.

Since the performance of the supervised model was strongly correlated with the size of the training data, the stability analysis of GAEDGRN was performed. We selected 10%, 20%, 30%, 40%, 50%, and 60% of all TFs+500 datasets with cell-type-specific ChIP-seq networks as a training set for stability analysis. Our model was run 10 times on each dataset. The results of the experiment are shown in Fig. 7. The results show that although the performance improves as the size of the training set increases, the dataset reaches a steady state when the training data reach about 50% and 60%. As a result, it turns out that GAEDGRN achieves praiseworthy results even in a small number of sample training datasets.

### Evaluation of computational complexity

In this section, in order to study the computational complexity, we compared the average running time of GAEDGRN with benchmark methods on seven scRNA-seq datasets. Table 1 shows the average runtime of each method on an NVIDIA GeForce RTX 3060 on seven single-cell datasets of different sizes for cell type-specific networks. Our method has the second shortest run time on TFs+500 genes and TFs+1000 genes scRNA-seq datasets. It is worth mentioning that when considering the computational complexity of these inference algorithms, our method demonstrates higher accuracy and stability in inferring GRNs across datasets of varying sizes (from small to large). In addition, our algorithm not only exhibits relatively short runtime, but also demonstrates relatively efficient performance.

**Table 1:** The average runtime for each method

Average running time	CNNC	DeepTFni	GNE	DGRNS	STGRNS	GAEDGRN
TFS+500 genes	30 min 40s	37 s	3 min 18 s	13 h 45 min	1 min 7 s	46 s
TFS+1000 genes	50 min 05 s	1 min 28 s	28 min 6 s	26 h 17 min	3 min 16 s	1 min 11 s

### Case study

**Figure 8:**
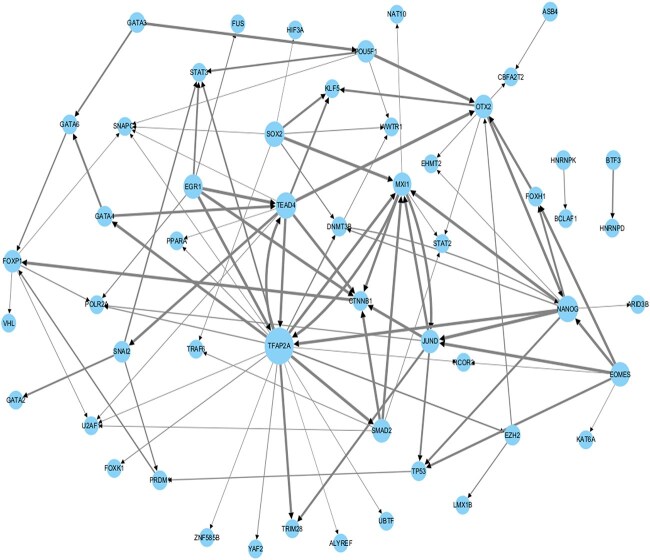
The top 100 edges with the highest scores in the cell-type-specific hESC TFs+1000 dataset are visualized, in which the larger the out-degree of the node, the larger the node, and the larger the edge score, the thicker the edge.

In this section, we performed a case study to further evaluate the predictive ability of GAEDGRN to recognize potential gene pairs. We performed this experiment on the cell-type-specific hESC TFs+1000 dataset. We first trained the model using the entire training set, saved the optimal model, and then used the optimal model to predict the entire GRN on the cell-type-specific hESC TFs+1000 dataset. Specifically, we used trained GAEDGRN to predict unknown gene pairs in specific hESC TFs+1000. Figure 8 shows the subnetwork of the top 100 predicted gene pairs, with *TFAP2A*, *TEAD4*, and *NANOG* being the three genes that have the largest number of out-degrees. *TFAP2A* is the encoded protein that can function as a homodimer or a heterodimer with similar family members. This protein activates the transcription of some genes while inhibiting the transcription of others. Defects in this gene are one of the causes of branchial facial syndrome [40]. *TEAD4*, an important member of the *TEAD* family and a downstream effector of the Hippo pathway, plays a crucial role in cell proliferation, survival, tissue regeneration, and stem cell maintenance. Many studies have shown that *NANOG* enhances specific characteristics of cancer stem cells and may therefore act as an oncogene to promote carcinogenesis [41]. As one of the key pluripotent TFs, *NANOG* plays a vital role in maintaining the self-renewal and pluripotency of normal embryonic stem cells. *NANOG* expression determines not only the cellular fate of pluripotent cells, but also the fate of cancer cells [42]. Therefore, we have predicted the target genes of the TFs *TFAP2A*, *TEAD4*, and *NANOG*. Panels A, B, and C of Supplementary Figs. S3 show the top 20 potential target genes sorted by the predicted scores through *TFAP2A*, *TEAD4*, and *NANOG,* respectively. We then manually validated the top 20 predicted target genes for *TFAP2A*, *TEAD4*, and *NANOG* in the Harmonizome database [43]. Of the top 20 predicted target genes for *TEAD4* 18 have been experimentally validated, 17 of the top 20 predicted target genes for *TFAP2A* have been experimentally validated, and 16 of the top 20 predicted target genes for *NANOG* have been experimentally validated. A detailed list of these validated genes is presented in Supplementary Table S4, and these results illustrate the effectiveness of GAEDGRN in predicting novel potential target genes for TFs.

## Conclusion

GRN is an important tool for elucidating gene function, interpreting biological processes, and analyzing complex markers of disease [44]. In this paper, we propose a GAEDGRN model for accurate inference of GRN from scRNA-seq data. GAEDGRN utilizes the GIGAE framework as its foundation, which not only enables the construction of directed GRN but also effectively extracts the structural features characteristic of directed networks. Secondly, we assumed and found that the higher the gene importance, the higher the likelihood that the gene would be linked to another unknown genes. To achieve the aforementioned assumption, we integrate the gene importance score matrix with the gene expression matrix to focus more on genes with high importance in the GIGAE encoder. To increase the likelihood of highly important genes being connected with other genes, we fused the gene scores with the mass parameters. Finally, to mitigate the issue of uneven distribution of latent vectors caused by GAE, we adapt random walk regularization to regularize the learning of the encoder. The experimental results demonstrate that our model achieves higher accuracy in GRN inference on the majority of datasets compared with the comparison models. Additionally, our model exhibits advantages such as good stability across datasets of varying sizes and low time complexity.

Although GAEDGRN performs well, there are still some shortcomings. Firstly, the disadvantage of supervised learning in biological network inference is that there are no reliable negative samples. In this case, we employ a random negative sampling strategy in which unknown gene pairs are randomly selected as negative samples. However, there is still the possibility of introducing false negatives. Secondly, the dataset we used is a static dataset, which contains less information about the regulatory relationships between genes than a time series dataset. GAEDGRN will ignore temporal dynamic information if it is directly scaled to a time series dataset. Therefore, in the future, we expect to use time series datasets and develop methods to infer more accurate GRNs.

**Key Points**

- This paper introduces GAEDGRN, a novel deep learning framework that employs GIGAE for directed GRNs inference based on single-cell RNA sequencing data.
- GAEDGRN innovatively incorporates the random walk method into GIGAE, in which the random walk regularization mechanism is employed to regularize the latent embeddings learned by the encoder.
- GAEDGRN introduces a novel approach to calculate the importance scores of genes, which can be used to predict novel potential target genes for TFs effectively.
- GAEDGRN outperforms the vast majority of state-of-the-art methods on datasets involving scRNA-seq data of seven cell types and three kinds of networks.

## Supplementary Material

Supplementary_Notes_bbaf232

## Data Availability

All the codes and datasets are available at https://github.com/jhjsagcdjks1/GAEDGRN.
