# Supplementary material for: GAEDGRN: reconstruction of gene regulatory networks based on gravity-inspired graph autoencoders
Source: Brief Bioinform. 2025 May 26;26(3):bbaf232. doi: 10.1093/bib/bbaf232 (PMC12104625; doi:10.1093/bib/bbaf232)
Supplement: Supplementary_Notes_bbaf232 [file supplementary_notes_bbaf232.pdf]

GAEDGRN：Reconstruction of gene regulatory networks based on gravity-inspired graph autoencoders

1 Supplementary Text

Performance evaluation metrics

This work uses area under the receiver operating characteristic curve (AUROC) and area under the precision-recall curve (AUPRC) as performance metrics for the model.

AUROC refers to the area under the receiver operating characteristic (ROC) curve. The ROC curve is a graphical representation of the true positive rate (TPR, see Equation 1) on the vertical axis and the false positive rate (FPR, see Equation 2) on the horizontal axis. The TPR is the proportion of positive samples correctly identified by the model, while the FPR is the proportion of negative samples incorrectly identified as positive. The closer the AUROC value is to 1, the better the classification performance of the model.

$$TPR = \frac{TP}{TP + FN} \tag{1}$$

$$FPR = \frac{FP}{FP + TN} \tag{2}$$

AUPRC refers to the area under the Precision-Recall curve. Precision (see Equation 3) refers to the ratio of true positive samples to all samples predicted as positive, while recall (see Equation 4) indicates the ratio of true positive samples to all actual positive samples. The closer the AUPRC value is to 1, the better the model's performance in balancing precision and recall, particularly in scenarios with limited positive samples.

$$Precision = \frac{TP}{TP + FP} \tag{3}$$

$$Recall = \frac{FP}{TP + FN} \tag{4}$$

2 Supplementary Tables

Table. S1 Pseudocode of the Node2Vec algorithm

Node2Vec with Restarts
Input: start node *v0*; path length *t*; restart probability *α*; Return *p*; In-out *q*
Output: path
1: path = [*v0*]
2: while length(path) < *t* do
3: curr = last node in path
4: rand = Random(0, 1)
5: if rand >= *α* then
6: neighbors = GetNeighbors(curr)
7: if length(neighbors) > 0 then
8: if length(path) == 1 then
9: next_node=RandomChoose(neighbors)

Table. S2 Pseudocode of the Node2Vec algorithm (Continued)

```
            else
                prev = second last node in path
                next_node = CalculateNextNode(curr, prev, p, q)
            end if
            AddToPath(path, next_node)
        else
            break
        end if
    else
        AddToPath(path, v0)
    end if
end while
return path
```

Table. S3 Pseudocode with random walk regularization

Regularization through Random Walk for Graph Autoencoder

```
Input: Graph G (V, X, A); window size w; walks per epoch γ; walk length t;
restart probability α; Return p; In-out q
Output: Gene embedding matrix Z′, reconstructed GRN Â
Z′ ← Encoder(G)
V′ = shuffle(V)
O = sample γ vertices from V′
for each v_i ∈ O do
    W_{v_i} = Node2Vec(A, v_i, t, α, p, q)
    for each v_j ∈ W_{v_i} do
        for each u_k ∈ W_{v_j}[j-w : j+w] do
            L_{v_j} = -logP(u_k | Z′(v_j))
    Update Skip-Gram and Encoder using ∇L_{v_j}
Z′ = Encoder (X, A)
Â ← Decoder (Z′)
return Z′, Â
```

Table. S3. Benchmark datasets

Cell types	Cells	STRING		Non-Specific		Specific	
		TFs	Genes	TFs	Genes	TFs	Genes
hESC	758	343(351)	511(695)	283(292)	753(1138)	34(34)	815(1260)
hHEP	425	409(414)	646(874)	322(332)	825(1217)	30(310)	874(1331)
mDC	383	264(273)	479(664)	250(254)	634(969)	20(21)	443(684)
mESC	421	495(499)	638(785)	16(522)	890(1214)	88(89)	977(1385)
mESC-E	1071	151(161)	291(413)	144(147)	442(674)	29(33)	691(1177)
mHSC-L	889	92(100)	201(344)	82(88)	297(526)	22(23)	618(1089)
mHSC-GM	847	39(40)	70(81)	35(37)	164(192)	16(16)	525(640)

Table. S4. GAEDGRN predicted data of the top 20 target genes of *TFAP2A*, *TEAD4*, and *NANOG* on specific hESC TFs+1000

Target gene of TFAP2A	Validation	Target gene of TEAD4	Validation	Target gene of NANOG	Validation
SMAD2	Confirmed	OTX2	Unconfirmed	JUND	Confirmed
TEAD4	Confirmed	TFAP2A	Confirmed	MXI1	Unconfirmed
MXI1	Confirmed	CTNNB1	Confirmed	TFAP2A	Confirmed
GATA4	Confirmed	SNAI2	Confirmed	FOXH1	Confirmed
TRIM28	Confirmed	KLF5	Confirmed	TP53	Confirmed
STAT3	Confirmed	U2AF1	Confirmed	DNMT3B	Confirmed
EZH2	Confirmed	PPARA	Confirmed	EHMT2	Confirmed
POLR2A	Confirmed	SNAPC3	Confirmed	ARID3B	Confirmed
DNMT3B	Confirmed	KAT6A	Confirmed	NAT10	Confirmed
YAF2	Unconfirmed	TFDP1	Confirmed	ZNF585B	Confirmed
FOXK1	Confirmed	EPHA2	Confirmed	TCOF1	Unconfirmed
PPARA	Confirmed	ZNF585B	Unconfirmed	FOXN3	Confirmed
WWTR1	Confirmed	HELZ	Confirmed	DNMT1	Confirmed
SNAPC3	Confirmed	ADSS	Confirmed	MRPS30	Unconfirmed
U2AF1	Unconfirmed	ZBTB38	Confirmed	NBPF14	Unconfirmed
UBTF	Confirmed	E2F4	Confirmed	MND1	Confirmed
ZNF585B	Confirmed	PPIC	Confirmed	SUPT7L	Confirmed
RCOR3	Confirmed	SNUPN	Confirmed	PBX1	Confirmed
ALYREF	Confirmed	FTSJ3	Confirmed	SPRY2	Confirmed
TRIM27	Unconfirmed	NOB1	Confirmed	RBM14	Confirmed

3 Supplementary Figures

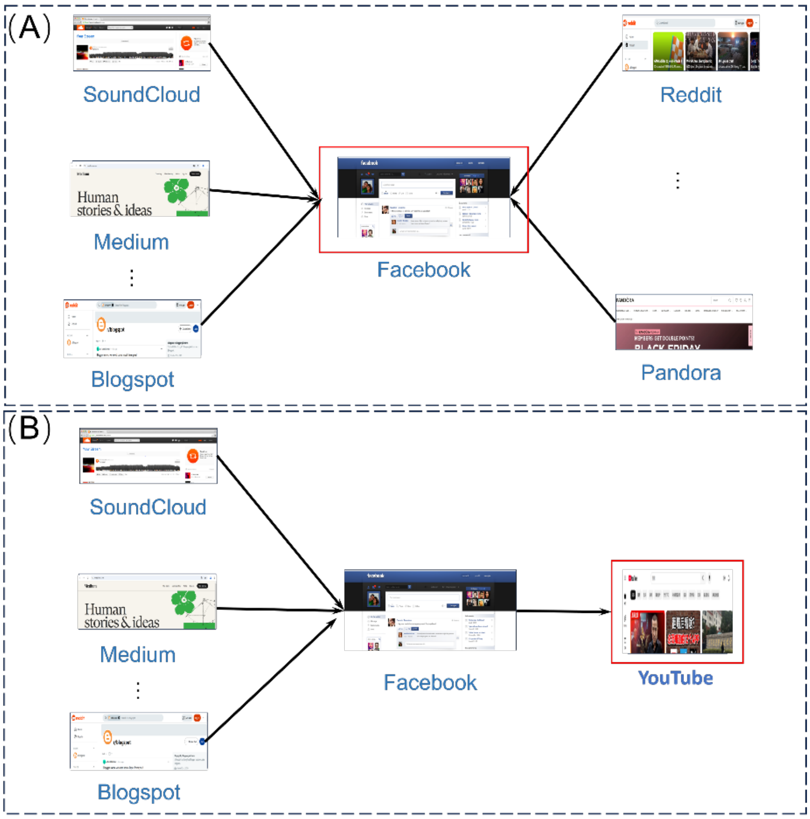

Fig. S1. There are two assumptions of the PageRank algorithm: (A) quality assumption (B) quantity assumption. The PageRank algorithm was originally designed to assess the importance of a web page, and its core idea is to calculate based on the entry degree of a node. The algorithm proposes two hypotheses: the quantity assumption and the quality assumption. Quantity assumption: If a page has many pages pointing to it, then the page is more important, such as: google, Facebook, etc. Quality assumption: If a page that is important points to a page, it is considered to be a page of high importance.

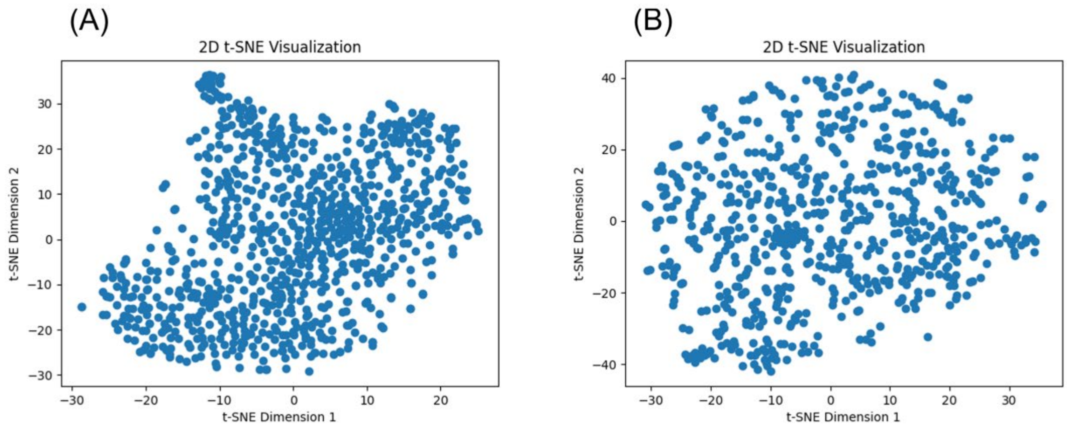

Fig. S2. (A) Latent vector distribution of genes without random walk regularization and (B) Latent vector distribution of genes with random walk regularization.

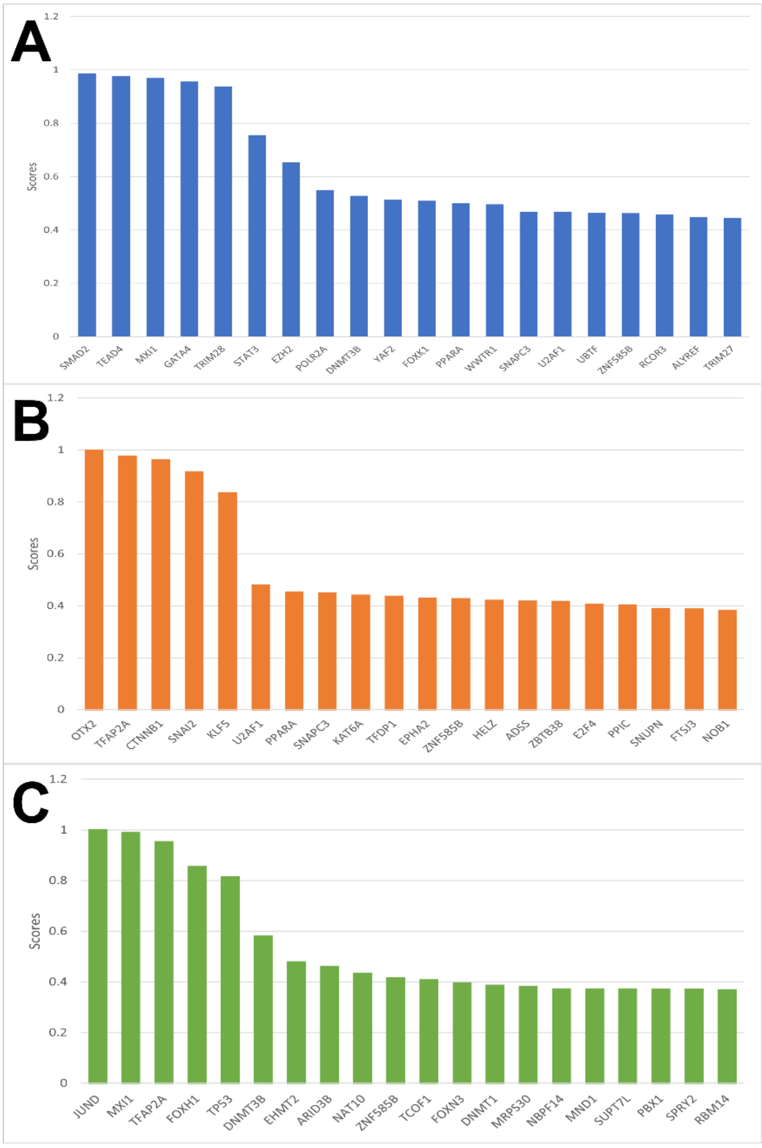

Fig. S3. (A), (B) and (C) are the ranking charts of the top 20 predictive potential target genes of genes *TFAP2A*, *TEAD4*, and *NANOG*, respectively.
